# Lymph Node Abscessation Secondary to Neoplasia in Two Dogs

**DOI:** 10.1155/2022/4726370

**Published:** 2022-03-26

**Authors:** Erin Close, Sarah Lumbrezer-Johnson, Eric Hostnik, Rhonda Burge, Ryan Jennings, Laura Selmic

**Affiliations:** ^1^The Ohio State University College of Veterinary Medicine, Department of Veterinary Clinical Sciences, USA; ^2^Ohio State University College of Veterinary Medicine, Department of Clinical Sciences, USA; ^3^The Ohio State University College of Veterinary Medicine, Department of Veterinary Biosciences, USA

## Abstract

A 5-year-old male neutered mixed breed dog and an 8-year-old female spayed golden retriever presented for cervical swelling which was later diagnosed as abscessation of the retropharyngeal lymph node with a malignant round cell tumor and carcinoma with multifocal squamous differentiation, respectively. In veterinary medicine, there is limited published information regarding abscessation of lymph nodes secondary to a neoplastic process. While more common in humans, there are only limited case reports available. Advanced imaging (computed tomography), cytology, surgical excision, and histopathology lead to the final diagnosis. Both dogs underwent surgical extirpation of the lymph nodes and adjuvant chemotherapy protocols. Six weeks postsurgical excision, dog one was euthanized due to quality-of-life concerns. The second dog successfully completed 18 treatments of radiation therapy and was still alive at 388 days postsurgical excision. At the time of manuscript submission, the second dog was doing well clinically.

## 1. Introduction

Cervical swelling in the canine is a common presenting complaint. Enquiries into the clinical history, the physical examination, and diagnostic imaging (radiographs and ultrasound) can aid in localizing the swelling [1]. Ultrasonography gives additional insight into the tissue of origin, tissue architecture, and vascular invasion and aids in obtaining a diagnostic sample through needle aspiration [1]. Cytological assessment allows identification of the predominant cell populations and further characterization of the swelling. Advanced imaging such as CT or MRI may be necessary for further characterization, identification of the tissue of origin, and surgical planning if applicable [1]. Benign and malignant disease processes can result in cervical swelling; as a result, the treatment of choice is variable dependent on cytological and histopathological diagnosis. Malignancy is among the top differentials for lymphadenopathy of the head and neck. Clinical presenting complaints in dogs are similar to that of humans, including cervical swelling, increased respiratory effort, anorexia, and lethargy. Differential diagnoses for lymphadenopathy can be assessed based on the number of lymph nodes affected and malignancy status. Single or regional lymphadenopathy can be differentiated into malignant causes (lymphoma or metastatic neoplasia) or nonmalignant causes which can be further differentiated into lymphoid hyperplasia, reactive lymphadenopathy, and lymphadenitis (skin infections, foreign bodies, periodontal disease, and rarely fungal infections) [2].

Lymphadenitis is defined as the presence of increased inflammatory cells within the lymph node and further defined by the predominant inflammatory cell type present [3]. Disseminated coccidioidomycosis, Coccidioides posadasii, was described in a canine with an oesophageal fistula and enlargement of the medial and lateral retropharyngeal lymph nodes [4].

Lymphadenopathy of multiple lymph nodes can be differentiated into malignant (lymphoma, metastatic solid neoplasia, lymphoid leukemia, and nonlymphoid leukemia) and nonmalignant causes as previously described; however, viral, bacterial, rickettsial, fungal, or protozoal infections, autoimmune diseases (systemic lupus erythematosus), or idiosyncratic drug reactions should be considered [2].

Lymphadenopathy secondary to metastatic neoplasia with associated abscessation is an uncommon presentation in humans and has never been reported in dogs with head and neck neoplasia to the author's knowledge. An abscess is a localized collection of purulent exudate [5]. A deep neck abscess in humans has been described as an abscess or infection deeper than the superficial layer of the deep cervical fascia [6.] Underlying factors can include dental disease, tonsillitis, trauma, foreign body penetration into the oral cavity, neoplasia, or unidentified causes, which represent 20-50% of cases in humans [4, 6, 7]. Cervical abscesses in the dog can similarly be associated with trauma, foreign body penetration or migration, dental disease, or iatrogenic introduction via fine needle aspirates.

In humans, diagnosis of neoplasia can be complicated by the presence of a coexisting infection [8]. Culture results of 90% of retropharyngeal abscesses in people had polymicrobial infections [7]. The diagnosis of an abscessed lymph node should not exclude neoplastic etiologies, especially where there is a known tumor. Misdiagnosis allows for continued tumor progression. A thorough physical examination, advanced imaging, and interpretations via cytology and histopathology are essential to obtain a diagnosis and a tailored treatment plan.

In the case report that follows, the authors describe the presentation, clinical findings, investigations, and outcome for two dogs that had abscessation of a lymph node secondary to neoplasia. This problem has never been reported in dogs with head and neck neoplasia and represents valuable learning opportunity about how to recognize and diagnose this condition.

## 2. Case Presentation

### 2.1. Case 1

A 5-year-old 21.9 kg (48 lb) neutered male mixed breed dog was evaluated at the Ohio State University Veterinary Medical Center (OSU-VMC) for cervical swelling, lethargy, and inappetence. Two days prior to presentation, the dog was evaluated by the primary care veterinarian for a several day history of increased stridor and panting at night. Additionally, the owners had noted an increase in serous nasal discharge. Swelling of the cervical region was described; however, no diagnostics were performed at this visit. The owner was instructed to monitor resting respiratory rate while at home and administer an antihistamine if the increased stridor and panting continued. The day prior to presentation at OSU-VMC, the dog appeared lethargic and had a decreased appetite. An antihistamine (unknown drug and dose) was given, and no improvement was appreciated. On presentation to OSU-VMC, the dog was bright, alert, and responsive. The dog was pyrexic with a rectal temperature of 103.9 F (reference interval (RI): 100-102.5 F) and approximately 5% dehydrated. A moderate nonpainful swelling of the cranial to midcervical region of the left side of the neck was identified. The left mandibular lymph node was markedly increased in size (measurements not available). Mild discomfort was elicited upon opening the jaw.

### 2.2. Case 2

An 8-year-old 36 kg (79.2 lb) female spayed golden retriever was evaluated at OSU-VMC for lethargy and anorexia. Historically, the dog had a left total ear canal ablation (TECA) performed three years prior for chronic otitis externa. Since the owners adopted the dog, one month prior to the previously performed TECA, they had noted mild stridor when sleeping. Upon presentation, the dog was quiet, alert, and responsive. The dog was pyrexic with a rectal temperature of 103.6 F (RI: 100.0-102.5 oF), and a large 10 *cm* × 20 *cm*, firm, nonmobile, subcutaneous mass was palpated in the region of the left subauricular and mandibular region. Mild respiratory stridor was appreciated when the dog was panting or anxious.

### 2.3. Case 1

A serum biochemical analysis revealed a mild elevation in aspartate aminotransferase (AST) (98 IU/L; RI: 16-51). A complete blood count (CBC) revealed a stress leukogram (leukocytosis: 17.8 × 109/L; RI: 4.8-13.9) characterized by neutrophilia (16.0 × 109/L; RI: 2.6-10.8) and lymphopaenia (0.9 × 109/L; RI: 1.0-4.6). Packed cell volume and total protein revealed mild hemoconcentration (53%; RI: 35-45 and 7.2 g/dL; RI: 5.2.-7.1). Radiographs of the cervical spine and thorax were facilitated by the administration of methadone (0.2 mg/kg) and acepromazine (0.02 mg/kg) intravenously (IV). Cervical spine radiographs revealed lobular, rounded soft tissue structures which extended to the dorsal nasopharynx and ventral oropharynx in the region of the soft palate and ventral displacement of the dorsal pharyngeal wall by soft tissue in the retropharynx. Findings were consistent with tonsillar hyperplasia and retropharyngeal lymphadenopathy. Three-view thoracic radiographs revealed a 3.5 cm soft tissue mass dorsal to the carina on the right lateral view consistent with mild tracheobronchial lymphadenopathy. Supportive care was initiated with IV Plasmalyte at 1.5 times maintenance, ondansetron (0.3 mg/kg IV every 8 hours), and methadone (0.2 mg/kg IV every 6 hours). Telemetry was maintained and no abnormalities were observed. A skull and cervical computed tomography (CT) scan was planned under general anesthesia. The patient was anesthetized with methadone (0.2 mg/kg) and midazolam (0.2 mg/kg) IV, and prior to intubation, an oral examination was performed. A 3-4 cm left tonsillar mass was identified. Intubation of a 10 mm internal diameter cuffed endotracheal tube was achieved with the administration of propofol (4 mg/kg bolus IV). General anesthesia was maintained with 1%-3% isoflurane (vaporizer setting) in oxygen (1 L/min).

**Figure 1:**
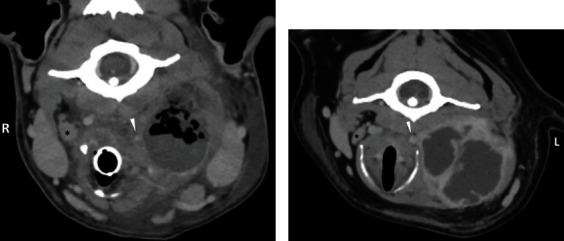
Transverse plane CT images of the left medial retropharyngeal lymph node abscesses. Case 1 (a) demonstrates severe lymphadenomegaly, with minimal peripheral contrast enhancement, and multiple intraluminal gas bubbles. Case 2 (b) demonstrates severe lymphadenomegaly, with peripheral contrast enhancement, as well as central septations that had similar contrast enhancement. Both cases have soft tissue stranding in the adjacent soft tissues and cause rightward deviation of the larynx. The contralateral medial retropharyngeal lymph node is labelled with an asterisk (∗). Notice that both medial retropharyngeal lymph nodes are bordered medially by the common carotid (arrowhead) and laterally by the mandibular salivary gland (cranially displaced by the mass in case 2).

The contrast CT (44 ml IV of iohexol 240 mg/ml) revealed an approximately 4.5 × 2.5 × 2.5 cm heterogenous, contrast-enhancing mass in the region of the left palatine tonsil with multiple, small gas bubbles immediately surrounding and in the periphery of the mass consistent with tonsillitis and or tonsillar abscessation. The left medial retropharyngeal lymph node was severely enlarged (7.0 × 3.5 × 4.5 cm) with minimal peripheral contrast enhancement and multiple gas bubbles, consistent with left medial retropharyngeal lymph node abscessation (Figure 1). The right medial retropharyngeal lymph node was moderately enlarged and rounded with irregular margins, suspected to be a reactive lymphadenopathy. Left-sided pharyngeal/laryngeal, left mandibular, and cervical subcutaneous edema was consistent with edema, cellulitis, myositis, or sialadenitis. The left and right medial retropharyngeal lymph nodes and the left mandibular lymph node were aspirated using ultrasound guidance. The left medial retropharyngeal lymph node was consistent with a septic suppurative inflammatory process; no overtly neoplastic cells were identified. A sample was additionally submitted for aerobic and anaerobic bacterial culture and antimicrobial sensitivity; Fusobacterium necrophorum was identified. The left mandibular and right medial retropharyngeal lymph nodes had rare, atypical cells, consistent with malignant neoplastic processes. Given the cellular characteristics, an anaplastic neoplasm was suspected. Due to significant concerns for respiratory distress following extubation, the left tonsillar mass was excised using a vessel sealing device (LigaSure, Medtronic). The mass was submitted for cytology and histopathology. An impression smear and cytology of the tonsillar mass revealed moderate lymphoid hyperplasia and mild to moderate suppurative inflammation with intracellular bacteria. Histopathology showed poorly differentiated malignancy. Following extubation, the dog recovered uneventfully and was started on carprofen (2.2 mg/kg subcutaneously (SQ) once), clindamycin (10.5 mg/kg IV every 12 hours), and enrofloxacin (10 mg/kg IV every 24 hours).

### 2.4. Case 2

A CBC revealed a leukocytosis (14.9 *X* × 109/L, RI: 4.8-13.9) characterized by a neutrophilia (13.4 × 109/L, RI: 2.6-10.8). A serum biochemical analysis was within normal limits. Prothrombin and partial thromboplastin times (PT/PTT) were within normal limits. Three-view thoracic radiographs revealed an unremarkable thorax. Fine needle aspirates of the left mandibular mass were nondiagnostic due to hemodilution. The dog was placed on IV Plasmalyte (at maintenance rate) and prescribed amoxicillin and clavulanic acid (13.75 mg/kg per os (PO) every 12 hours), gabapentin (10 mg/kg PO every 8 hours), trazodone (4 mg/kg PO every 8 hours), butorphanol (0.2 mg/kg IV every 6 hours as needed), and dexamethasone sodium phosphate (0.07 mg/kg IV once). A cervical/skull CT scan with contrast (60 mL of iohexol 240 mg/mL IV) was facilitated by the administration of dexmedetomidine (5 mcg/kg) and butorphanol (0.2 mg/kg) IV. There was a 6 cm round, cavitated, rim-enhancing soft tissue mass, located between the bifurcation of the left common carotid artery and the mandibular salivary gland which deviated the larynx, esophagus, and associated structures to the right (Figure 1). Considerations given to the cavitated mass included neoplasia, abscess, or lymphadenitis. Caudal to the left retropharyngeal mass was a separate soft tissue structure that was suspected to represent an enlarged caudally displaced left medial retropharyngeal lymph node or an enlarged cranially positioned deep cervical lymph node. The left mandibular lymph nodes were mildly enlarged and suspected to be metastatic or reactive. Ultrasound-guided aspirates of the mass and adjacent lymph nodes were performed. The cervical lymph node (lymph node not specified) was consistent with mild to moderate lymphoid hyperplasia, moderate to marked plasma cell hyperplasia, and mild mixed inflammation. Fine needle aspirates of the left retropharyngeal mass were concerning for squamous cell carcinoma, marked mixed inflammation, and necrosis. An abdominal ultrasound did not reveal any notable metastatic neoplasia.

### 2.5. Case 1

In the following day, the dog was placed under general anesthesia using a premedication of methadone (0.2 mg/kg) and midazolam (0.2 mg/kg) IV. This facilitated intubation with an 11 mm internal diameter cuffed endotracheal tube with a propofol (2.6 mg/kg bolus IV), and the dog was maintained under anesthesia with 1%-3% isoflurane (vaporizer setting) in oxygen (1 L/min) for surgical extirpation of the left and right mandibular and medial retropharyngeal lymph nodes. A Jackson-Pratt drain was placed prior to closure. Following recovery from surgery, the Jackson-Pratt drain was emptied and quantified every 6 hours or if greater than 50% full. The dog was monitored for signs of respiratory distress (increased respiratory rate and or increased respiratory effort). The dog received methadone (0.2 mg/kg IV every 6 hours) and carprofen (2.2 mg/kg SQ once), warm compresses were applied to the neck following the procedure every 8 hours, and enrofloxacin and clindamycin were continued. One-day postoperatively, the dog was transitioned to oral medications (gabapentin 10 mg/kg PO every 8 hours, carprofen 2.2 mg/kg PO every 12 hours, clindamycin 7 mg/kg PO every 12 hours, enrofloxacin 9.2 mg/kg PO every 24 hours, and trazodone 4.3 mg/kg PO every 8 hours as needed). The JP drains were removed, and the dog was discharged from OSU-VMC three days postsurgery with instructions to continue oral medications as described above.

### 2.6. Case 2

The dog received methadone (0.2 mg/kg) and acepromazine (0.03 mg/kg) IV as premedication and was placed under general anesthesia. Prior to intubation, an oral examination was performed and revealed a left-sided tonsillar mass (measurements not available). Intubation of a 12 mm internal diameter cuffed endotracheal tube was achieved with the administration of propofol (3 mg/kg bolus) IV, and the dog was maintained with 1%-3% isoflurane (vaporizer setting) in oxygen (1 L/min). The left retropharyngeal mass was located and excised from caudal to cranial using blunt dissection, with care taken to avoid the recurrent laryngeal nerve, vagosympathetic truck, and carotid artery. The mass was intimately associated with the left jugular vein, and consequently, this was ligated. The left retropharyngeal lymph node was carefully dissected and excised. Upon gross examination, the mass was cavitated, red to dark purple in color with a firm white capsule and yellow to white pus was evident on cut surface. A left tonsillectomy was also performed for concerns of respiratory distress following extubation. The tonsillar crypt was closed with a simple continuous pattern. A sample was additionally submitted for aerobic and anaerobic bacterial culture and antimicrobial sensitivity from the left medial retropharyngeal lymph node; no growth was identified. A Jackson-Pratt drain was placed prior to closure and secured with 2-0 Monosoft. The dog was monitored for signs of respiratory distress (increased respiratory rate or effort). Following recovery from surgery, the Jackson-Pratt drain was emptied and quantified every 6 hours, or if greater than 50% full. Facial swelling was massaged for 5-10 minutes every 8 hours. The dog was placed on Plasmalyte (maintenance), ampicillin and sulbactam (30 mg/kg IV q 8 hours), methadone (0.1 mg/kg IV q 6-8 hours as needed for pain), meloxicam (0.1 mg/kg SQ once), and gabapentin (8.3 mg/kg PO q 8 hours). One day postsurgery, the ampicillin and sulbactam was transitioned to oral amoxicillin and clavulanic acid (14 mg/kg PO q 12 hours), and injectable meloxicam was transitioned to oral (0.1 mg/kg PO q 24 hrs). Three days postoperatively, the JP drain was removed, and the dog was discharged with instructions to continue amoxicillin and clavulanic acid (14 mg/kg PO q 12 hours), meloxicam (0.1 mg/kg PO q 24 hours), gabapentin (8.3 mg/kg PO q 8 hours), and trazodone( 4.2 mg/kg PO q 8-12 hours as needed for anxiety).

### 2.7. Case 1

**Figure 2:**
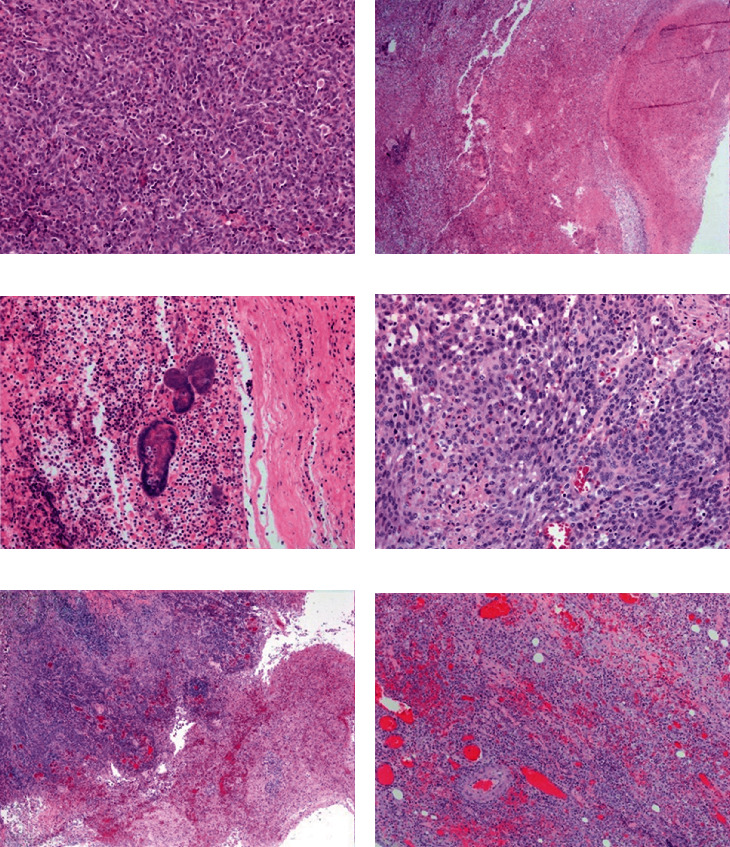
Histopathology of case 1 (a–c) and case 2 (d–f). In case 1, the neoplastic round cells (a) effaced the lymph node architecture with regions of lytic necrosis (b) associated with bacterial colonies and suppurative inflammation (c). In case 2, the neoplastic epithelial cells (d) effaced the lymph node with large central regions of lytic necrosis € as well as abundant pyogranulomatous inflammation and granulation tissue, elsewhere (f).

Histopathology of the left tonsillar mass and right and left medial retropharyngeal and mandibular lymph nodes were consistent with poorly differentiated malignancy that required additional staining that was consistent with a malignant leukocytic neoplasm (expression of CD18 and CD45) (Figure 2(a)). Neoplastic cells were immunonegative for CD3, CD20, cytokeratin, and SOX10, with equivocal Iba-1 labeling. The left retropharyngeal lymph node additionally had severe necrotizing lymphadenitis with intralesional bacteria (Figures 2(b) and 2(c)). Two weeks postsurgery, the patient presented to OSU-VMC for a postoperative recheck. At this visit, blood work and further staging were performed. A CBC revealed a grade II thrombocytopenia (94 × 109/L; RI: 145-463) [9]. A biochemical analysis was within normal limits. An abdominal ultrasound was facilitated by butorphanol 0.2 mg/kg and acepromazine 0.03 mg/kg intramuscularly (IM) and revealed a splenic mass with multicentric cranial abdominal lymphadenopathy. Fine needle aspirates of the spleen and perihepatic lymph nodes were performed following IV administration of dexmedetomidine (5 mcg/kg). Cytology of the perihepatic lymph node and splenic mass was consistent with malignant discrete cell neoplasia. Considerations were given to histiocytic sarcoma (given the CD18 positive expression previously noted), atypical lymphoma, melanoma, and anaplastic sarcoma. A tentative diagnosis of tonsillar and metastatic histiocytic sarcoma was made considering the clinical pathology, histopathology, and immunochemistry results. Given significant concern for disseminated histiocytic sarcoma, lomustine (CCNU 61.4 mg/m2 PO) and maropitant citrate (1 mg/kg PO) were administered at this time. The dog was prescribed prednisone (1 mg/kg PO every 24 hours), maropitant citrate (2.7 mg/kg PO every 24 hours), ondansetron (0.7 mg/kg PO every 8-12 hours), metronidazole (11 mg/kg PO as needed for diarrhea), and amoxicillin and clavulanic acid (17 mg/kg PO every 12 hours for 10 days).

Five weeks postsurgery, the dog was evaluated again. Progressive disease was identified by enlargement of a suspected right mandibular lymph node (1.5 cm). An aspirate of the lymph node confirmed metastatic disease. A CBC revealed a progressive grade II thrombocytopenia (71 × 109/L; RI: 145-463) and leukopenia (2.8 × 109/L; RI: 4.8-13.9), characterized by grade I neutropenia (1.9 × 109/L; RI: 2.6-10.8). The dog was continued on the previously prescribed prednisone, maropitant citrate, ondansetron, and metronidazole.

Another evaluation was performed six weeks postsurgery; progressive peripheral lymph node enlargement was identified (mandibular, prescapular, axillary, and popliteal). A CBC revealed a grade IV thrombocytopenia (22 × 109/L; RI: 145-463) and grade II neutropenia (1.4 × 109/L, RI: 2.6-10.8), thought to be due to metastatic involvement of the bone marrow [9]. Thoracic radiographs revealed progressive tracheobronchial and sternal lymphadenopathy. The dog was suspected to have lymphoma at that time. Vincristine (0.6 mg/m^2^) was given IV. The previously prescribed prednisone, maropitant citrate, ondansetron, and metronidazole were continued. At 8 weeks postoperatively, the dog was euthanized due to decreased quality of life and advanced disease.

### 2.8. Case 2

Surgical biopsy of the left retropharyngeal mass was consistent with an unencapsulated, invasive, epithelial neoplasm. Neoplastic cells were arranged in thick anastomosing cords, trabeculae, and nests radiating away from a luminal structure into a densely collagenous stroma consistent with a carcinoma with multifocal squamous differentiation (Figure 2(d)). Origination from the salivary gland ductal epithelium was suggested due to the appearance of the carcinoma surrounding a lumen that contained abundant inflammatory debris within the lumen (Figure 2(e)). Additionally, extensive pyogranulomatous steatitis and fasciitis with multifocal myofiber degeneration and necrosis surrounded the neoplasm (Figure 2(f)). The left tonsil was consistent with moderate lymphoid follicular hyperplasia.

Upon reexamination, six days postsurgery, mild necrosis of the edges of the proximal 2/3 of the incision was identified. No discharge was present at this time. The incision was managed with a tie over bandage until primary closure could be pursued. Culture of the draining tract grew methicillin-resistant Staphylococcus pseudintermedius (MRSP). Based on sensitivity results, the amoxicillin and clavulanic acid was discontinued and chloramphenicol (41 mg/kg PO every 8 hours) was initiated. The wound was closed primarily 15 days postsurgery. A neck CT was performed under general anesthesia (butorphanol (0.2 mg/kg) and dexmedetomidine (3 mcg/kg) IV, followed by propofol (3 mg/kg bolus) IV and maintained with 1%-3% isoflurane (vaporizer setting) in oxygen (0.8 L/min)) in preparation for definitive radiation therapy. At the previous surgical site, residual edema, fibrosis, and/or cellulitis were identified; however, residual, direct extension of the mass with irregular tendril-like growth could not be excluded. 42 days postsurgery, the dog started definitive radiation therapy and was placed under general anesthesia once a day for eighteen days (propofol 6-6.5 mg/kg bolus IV and maintained with 2.5-4% sevoflurane (vaporizer setting) in oxygen 1-3 L/min), receiving 2.67 gray (Gy) daily to the previous surgical site with 6MV photons using intensity-modulated radiation therapy (IMRT). Piroxicam (0.3 mg/kg PO every 24 hours) was prescribed to help reduce inflammation associated with radiation. During the third week of radiation therapy, the dog developed desquamation of the skin in the region of the neck; topical amikacin 1% spray (spray affected area of the neck once daily) was prescribed. Due to concerns of radiation induced esophagitis, omeprazole (0.6 mg/kg orally every 12 hours) and sucralfate (30 mg/kg PO every 8 hours, 1-2 hours prior to or after food or other medications) were prescribed. Eighty days postsurgical excision, a CBC and serum biochemical analysis were performed and were clinically unremarkable. The first dose of carboplatin (300 mg/m^2^ IV) was administered. Additionally, maropitant citrate (1 mg/kg) was given IV once prior to carboplatin administration. Maropitant citrate (2.5 mg/kg PO every 24 hours for 4 days), ondansetron (0.5 mg/kg PO every 8-12 hours as needed for nausea, vomiting, or inappetence), and metronidazole (11 mg/kg PO every 12 hours as needed for diarrhea or soft stool) were prescribed as needed for supportive therapy. The dog became anorexic (grade III) for the first 3-4 days following the administration of the first dose of carboplatin [9]. Ninety-one days postsurgical excision, the dog had a CBC which revealed a leukopenia (1.5 × 109/L, RI: 4.8-13.9), characterized by grade IV neutropenia (0.2 × 109/L; RI: 2.6-10.8) and grade III thrombocytopenia (25 × 109/L; RI: 145-463) [9]. Amoxicillin and clavulanic acid (14 mg/kg PO every 12 hours) was initiated. Ninety-two days postsurgical excision, the dog presented to the OSU-VMC Emergency Service for an episode of vomiting and diarrhea. Upon presentation, the dog was bright, alert, and responsive. The dog was pyrexic with a rectal temperature of 103.0 F (RI: 100.0-102.5 F) and euhydrated. At this time, hospitalization was recommended for supportive care and further diagnostics; however, the owners elected to monitor at home for worsening of clinical signs. Ninety-three days postsurgical excision, the dog represented to the OSU-VMC Emergency Service for continued vomiting (grade 2), diarrhea, anorexia, and lethargy [9]. Upon presentation, the dog was dull but responsive. The dog was pyrexic with a rectal temperature of 104.3 F (reference interval 100.0-102.5 F) and estimated to be 5-7% dehydrated. Packed cell volume and total protein revealed mild hemoconcentration (42%; RI: 35-45 and 8.8 g/dL RI: 5.2.-7.1). A CBC revealed a progressive leukopenia (0.24 × 109/L, RI: 4.8-13.9), characterized by grade V neutropenia (0.01 × 109/L; RI: 2.6-10.8) and grade IV thrombocytopenia (0 × 109/L; RI: 145-463) [9]. A manual platelet count revealed 3-5 platelets per high powered field. The dog was hospitalized on Plasmalyte (100 mls/hr), ondansetron (0.2 mg/kg IV every 8 hours), maropitant citrate (1 mg/kg IV every 24 hours), pantoprazole (1 mg/kg IV every 12 hours), ampicillin and sulbactam (30 mg/kg IV every 8 hours), and metronidazole (15 mg/kg IV every 12 hours). A nasogastric tube was placed, and the dog was fed 1/3 of her resting energy requirement with Clinicare. In the following day (94 days postsurgical excision), Yunnan Baiyao (500 mg orally every 8 hours) was initiated due to continued epistaxis from the placement of the nasogastric tube.

Recheck blood work 95 days postsurgery later revealed mild improvement in the leukopenia (0.37 × 109/L, RI: 4.8-13.9), with static grade V neutropenia (0.01 × 109/L; RI: 2.6-10.8) and grade IV thrombocytopenia (0 × 109/L, RI: 145-463) [9]. The PCV revealed moderate anemia (28%; RI: 35-45%). At this time, the dog was pancytopenia, and enrofloxacin (10 mg/kg IV every 24 hours) was started. Ninety-nine days postsurgery, the dog was discharged from the hospital after continued increases in leukocytes and platelets. At this time, the owners elected to discontinue the carboplatin chemotherapy protocol and treat symptomatically with prednisone (0.5 mg/kg PO every 24 hours). One hundred and six days postsurgery, the dog returned for a CBC which revealed a significantly improved leukopenia (10.9 × 109/L, RI: 4.8-13.9), neutropenia (8.0 × 109/L; RI: 2.6-10.8), and thrombocytopenia (192 × 109/L, RI: 145-463). The PCV was mildly decreased (33%; RI: 35-45%). Piroxicam (0.3 mg/kg orally every 24) was prescribed to the dog. One hundred and ninety-eight days postsurgery, 3-view thoracic radiographs showed no evidence of metastatic neoplasia, and CBC and serum biochemical analysis were unremarkable. Two hundred ninety-seven days postsurgery, the dog was sedated (dexmedetomidine 3 mcg/kg and butorphanol 0.2 mg/kg IV) for a repeat cervical CT and 3-view thoracic radiographs. These did not reveal evidence of metastatic neoplasia or recurrence of the mass. CBC and serum biochemical analysis were unremarkable. Three hundred eighty-eight days postsurgery, recheck 3-view thoracic radiographs, CBC, and serum biochemical analysis were clinically normal.

## 3. Discussion

In veterinary medicine, a single case report described abscessation associated with an intramuscular hemangiosarcoma in a dog; to the author's knowledge, this is the only report of abscessation and associated neoplasia in the dog [10]. Initial presentation of cervical cellulitis or a deep neck abscess as a result of head and neck malignancy is rare in humans [11]. Often in humans, squamous cell carcinoma of the head and neck has cervical metastases at the time of diagnosis. Cystic nodal metastases may arise from the nasopharynx, tonsil, or thyroid gland [12]. Abscessation has been hypothesized to occur from infection of the cystic metastases, as a result of central necrosis of the tumor, or direct tumor extension that has undergone necrosis [12]. Trauma of the underlying skin with subsequent infection or iatrogenic introduction of bacteria from diagnostic sampling is additional potential explanations [10].

In evaluating human patients, a deep neck abscess should be considered a differential diagnosis if severe, acute pain is reported, upper respiratory tract infection, trauma associated with the head or mouth, respiratory distress, dysphagia, asymmetry of the neck, lymphadenopathy, cranial nerve involvement, pyrexia, tachypnoea, or immunosuppression [7]. In human medicine, lymph node abscesses have been associated with nasopharyngeal carcinoma, Hodgkin's lymphoma, B-cell lymphoma, squamous cell carcinoma, and carcinoma of unknown primary [8, 11–13]. Both dogs presented to the teaching hospital for lethargy, hyporexia to anorexia, and stridor while at rest. Dog 1 additionally presented for cervical swelling. Upon performing physical examinations, both dogs were pyretic, cervical swelling was observed in dog 2, and discomfort elicited upon movement of the temporomandibular joint in dog 1. Neither dog had a history of trauma associated with the head and neck; however, dog 2 had a TECA on the same side of the cervical swelling three years prior.

Advanced imaging, such as CT, is the preferred imaging modality for diagnosis of deep cervical abscess in humans; however if the patient is not stable enough, cervical radiographs can be performed [7]. Only dog 1 had cervical radiographs taken, identifying tonsillar hyperplasia, retropharyngeal lymphadenopathy, and tracheobronchial lymphadenopathy. Advanced imaging (CT) was not pursued at the time of presentation given the stability of the dogs and the availability of CT at our institution. Due to the proximity of the deep neck with the airway, cervical vessels, and the spinal cord, evaluating the extent of the abscess is vital [14]. Mediastinitis or empyaema can be sequela in advanced disease [7]. Both dogs in this report had thoracic radiographs performed which did not identify these abnormalities. In addition to CT, MRI can be used to further evaluate if medial retropharyngeal lymph node mass lesions in dogs and cats are neoplastic or inflammatory lymphadenitis in origin. Mass inflammatory in origin was more likely to have moderate or marked MRI perinodal and local muscle contrast enhancement, while neoplastic masses in origin were more likely to have larger MRI width and height [15].

A skull and cervical spine CT was performed in both cases. Case 1 had an oral examination performed prior to intubation which revealed a left-sided tonsillar mass. In case 2, the skull and cervical CT was performed under sedation, without an oral examination. The dog in case 2 received an oral examination prior to being induced for surgery, where a left tonsillar mass was identified. A thorough oral examination, prior to advanced imaging when possible, is an important part of the evaluation in cases with cervical swelling. Based on CT findings of the tonsillar and left medial retropharyngeal lymph node in Case 1, a heterogenous soft tissue, contrast-enhancing mass that was only peripherally enhancing in the lymph node, with multiple gas bubbles immediately surrounding and in the periphery of the masses, was suspicious of abscessation of the left tonsil and left medial retropharyngeal lymph node. In case 2, contrast enhancement with gas was not identified; however, the mass was cavitated and rim enhancing with contrast. Considerations were given to neoplasia, abscess, or lymphadenitis.

Due to concerns of abscessation, a fine needle aspirate and bacterial culture with antimicrobial sensitivity of the retropharyngeal lymph node were performed in case 1. The results were consistent with septic suppurative inflammation with no overtly neoplastic cells identified and growth of Fusobacterium necrophorum. Cytology of regionally involved lymph nodes was consistent with a malignant neoplastic process. A tonsillar impression smear was consistent with lymphoid hyperplasia and suppurative inflammation. An additional bacterial culture and antimicrobial sensitivity were not performed at closure due to the complete excision of the abscess tissue focus, local lavage, and the placement of a closed suctioned drain. In case 2, aspirates of the left medial retropharyngeal lymph node and regionally affected lymph nodes were consistent with squamous cell carcinoma and moderate lymphoid and plasma cell hyperplasia, respectively. At the time of surgical excision, bacterial culture and antimicrobial sensitivity were submitted from the left medial retropharyngeal lymph node; however, no growth was identified. Given the gross appearance of purulent discharge on cut surface of the left medial retropharyngeal lymph node with a negative culture, there remained clinical suspicion that a component of abscessation was present. The presence of an abscess and infection further complicates the definitive diagnosis of malignancy due to the superimposition of these processes yielding a sample that is not representative [8]. While no growth was observed from the culture in case 2, the sample was collected from the excised lymph node. Local lavage and a closed suctioned drain were used prior to closure. However, the dog returned to OSU-VMC six days postoperatively with an incisional dehiscence an injection that required open wound management. It is unknown as to whether this was a result of local infection associated with the suspected abscess or a surgical site infection with MRSP.

Due to inconclusive cytology results in case 1, histopathology was performed on the tonsillar mass and regional lymph nodes. Based on histopathology alone, a definitive diagnosis could not be made. Immunohistochemistry of the samples was performed identifying the presence of CD18+ and Iba-1 markers, consistent with histiocytic sarcoma. Two weeks postoperatively, further staging was performed identifying metastatic histiocytic sarcoma within the spleen and cranial abdominal lymph nodes. In the canine, histiocytic sarcomas are one of the most aggressive and incurable tumors diagnosed [16]. Surgical intervention of histiocytic sarcomas alone may be adequate if metastasis is not detected at the time of diagnosis [17]. It has been suggested that if the histiocytic sarcoma is applicable, surgical intervention of localized disease, chemotherapy, and radiotherapy may increase median survival time [18]. Treatment of histiocytic sarcomas with sole CCNU chemotherapy protocols has shown tumor response rates varying between 29 and 46% [17]. In this case, the dog did not respond to treatment with CCNU due to progressive disease characterized by metastasis to the bone marrow, spleen, and cervical and abdominal lymphadenopathy. Given diffuse lymphadenopathy, lack of response to CCNU therapy, and concern for advanced lymphoma, the dog received vincristine as a rescue protocol. In humans with Langerhans cell histiocytosis, similar to disseminated disease in canines, vinca alkaloids can be effective. The combination of select bisphosphonates (zoledronate and clodronate) with vinca alkaloids may increase the overall effectiveness of the chemotherapy agents on disseminated histiocytic sarcomas though increased tumor cell permeability and cell cycle arrest [19]. However, due to the owners' wishes and poor quality of life, the patient was euthanized. In case 2, a definitive type of neoplasia could not be diagnosed via histopathology; only cytologic diagnosis of suspected squamous cell carcinoma was made. Furthermore, histopathology was unable to confirm the presence of an intralesional abscess. Based upon CT and gross findings, the clinicians were highly concerned for abscessation of the left medial retropharyngeal lymph node. Based on cytology results, a chemotherapy protocol with carboplatin and adjunctive piroxicam was initiated. Carcinoma overexpression of COX-2 enzymes allows increased tumor cell proliferation, resistance to apoptosis, and angiogenesis. Piroxicam inhibits the synthesis of prostaglandins through the selective inhibition of the COX enzyme. Platinum-containing chemotherapy drugs lead to inhibition of DNA synthesis and hinder DNA repair due to the large size of the molecule [20]. When used in combination to treat oral nontonsillar squamous cell carcinomas, a 57% response rate to complete remission was identified [21].

Information regarding abscessation of lymph nodes with associated neoplasia is limited in veterinary medicine. Further work-up of the cases reported was required when a response to the empirical treatment and monitoring was unsuccessful in resolving the problem. Advanced imaging, cytology, histopathology, and culture (bacterial and fungal) were pursued to rule out antibiotic resistance, mycobacterial or fungal infections, and neoplastic or inflammatory conditions [22]. Although potentially rare, abscessation with associated neoplasia should be considered as a differential diagnosis for cervical swelling in the canine patient.

## Data Availability

The data used to support the findings of this study are included within the article.
